# On the Role of ZrN Particles in the Microstructural Development in a Beta Titanium Alloy Processed by Laser Powder Bed Fusion

**DOI:** 10.3390/mi15010104

**Published:** 2024-01-05

**Authors:** Xu Chen, Chunlei Qiu

**Affiliations:** School of Materials Science and Engineering, Beihang University, Beijing 100191, China; chxuxc@buaa.edu.cn

**Keywords:** laser bed powder fusion, titanium alloy, inoculation particles, solidification behavior, columnar-to-equiaxed transition

## Abstract

Additive manufacturing of titanium alloys usually ends up with large columnar grains due to the steep thermal gradients within melt pools during solidification. In this study, ZrN particles were added into a beta titanium alloy, Ti-10V-2Fe-3Al, with the aim of promoting columnar-to-equiaxed grain transition during laser bed powder fusion (L-PBF). It was found that the addition of ZrN leads to the development of alternate layers of equiaxed grains and refined columnar grains, which is in sharp contrast to the dominant large columnar grains formed in the pure L-PBF-processed titanium alloy. An investigation on single laser melted tracks revealed that the sample with added ZrN showed fine equiaxed grains in the upper regions of solidified melt pools and columnar grains in the lower regions, whereas the solidified melt pools of the pure titanium alloy were dominated by large columnar grains due to epitaxial growth from the previous layer. The formation of equiaxed grains in the former sample is attributed to multiple factors including an increased gradient of liquidus temperature due to the solution of N and a reduced actual melt temperature gradient due to the melting of high-melting-point ZrN particles, which would have expanded constitutional undercooling, a grain growth restriction effect induced by the segregation of N along grain boundaries and the accumulation of unmelted ZrN particles in the upper regions of melt pools. The addition of ZrN also resulted in significant α precipitation, which showed strong variant selection and was found to be driven by laser reheating and the N solution in the matrix.

## 1. Introduction

Metallic additive manufacturing (AM) is a disruptive technology known for its outstanding near-net-shape manufacturing capability that offers great design flexibility and enables a high level of product customization. It is also known for its unique processing characteristics including complex power beam-material interaction [1,2,3,4,5], unstable melt flow behavior, high cooling rates and high thermal gradients in melt pools [6,7,8], and complex thermal cycling that can partially remelt or reheat previous solidified layers. A steep thermal gradient usually results in epitaxial grain growth while the remelting of previously solidified layers can eliminate the equiaxed grains that have formed in the upper region of a solidified layer. As a result, columnar grains are frequently observed in as-fabricated components, which are strongly associated with the development of hot tearing [9,10,11], chemical segregation [10,11] and mechanical property anisotropy [12,13,14,15]. A transition to a fine equiaxed grain structure brings many benefits to mechanical properties including improved strength, ductility, fatigue life and reduced crack susceptibility [9].

To promote columnar-to-equiaxed transition (CET) during metallic AM, one could resort to mechanical means such as in situ ultrasonic treatment [16], which, however, may involve a complicated setup of an additional facility in AM machines. One could also promote CET and grain refinement by increasing constitutional undercooling (CU or ∆*T*_CU_) ahead of the solidification front or reducing the critical amount of undercooling required for equiaxed grain nucleation (∆*T*_N_). ∆*T*_CU_ can be changed by changing the thermal conditions and alloy constitution while ∆*T*_N_ can be reduced by introducing potent nucleants into melt. Based on these fundamental principles for the CET, a number of attempts have been made to promote CET in a number of material systems during AM through either process control or the addition of nucleation particles. Zhang et al. [17,18] increased the tendency for CET in the laser direct energy deposition (L-DED) of Ti-6Al-2Sn-2Zr-3Mo-1.5Cr-2Nb by increasing the powder deposition rate and decreasing the laser energy density, which created heterogeneous nucleation on partially unmelted powder particles. Wang et al. [19] also reported this same observation during the L-DED of Ti-6.5Al-3.5Mo-1.5Zr-0.3Si, as did Wu et al. [20], who observed CET with decreased energy density during the L-DED of Ti-25V-15Cr-2Al-0.2C. With L-PBF, it is not straightforward to achieve a fully equiaxed grain structure by simply changing processing conditions, given that the melt pools generated during this process are even smaller than those produced using L-DED or wire-arc AM (WAAM) [21], which means that the thermal gradients in the melt pools created during these processes could be even steeper. Normally, with L-PBF, changing the processing parameters can lead to a change in the grain size and texture, but they could hardly lead to a full CET [22,23,24,25,26,27,28]. Qiu et al. [29] increased the powder layer thickness in the L-PBF of a β titanium alloy with the aim of acquiring a full equiaxed grain structure by creating larger melt pools and promoting heterogeneous nucleation on potential partially melted particles, but ended up with a hybrid columnar and equiaxed grain structure.

Alloy solutes with a large growth restriction factor, *Q*, which is defined by *mC*_0_(*k* − 1) where m is the slope of the liquidus line, *C*_0_ is the solute concentration in the bulk alloy and k is the equilibrium partition coefficient of solute in solid phase and liquid phase, can rapidly develop ∆*T*_CU_ and provide effective grain refinement [6]. Titanium alloys which contain solutes with a large *Q*, such as B [30,31,32,33,34], C [35], Cr [6], Si [36] and Cu [37], have been found to develop either a refined grain structure or a equiaxed grain structure during AM. This is in sharp contrast to those that do not contain such elements, such as Ti-6Al-4V, with which the CET is far harder to achieve during AM. Recently, Qiu et al. [38] further proposed that Fe and Co show a high *Q* value in titanium alloy, i.e., *Q*_Fe_ = 3.2 and *Q*_Co_ = 19.2, and thus could also induce CET during L-PBF. Based on the novel alloy design philosophy, they have successfully developed an Fe and Co-containing metastable beta titanium alloy which is dominated by fine equiaxed grains. Overall, adding elements with a high *Q* value is a promising route for promoting CET or grain refinement during the L-PBF process.

Another approach to promote the CET in additively manufactured metallic materials lies in the introduction of potent nucleating particles. This has proven particularly effective for the AM of Al-based metals via the addition of Al_3_Zr [9], Al_3_Sc [39], Al_3_Ta [40], TiB_2_ [41] and TiC [42] nucleants. In comparison, during the AM of Ti alloys, there is a lack of available potent nucleant particles and a lack of insight into what foreign particles can catalyze the heterogenous nucleation of β-Ti grains at a small ∆*T*_N_. Among the limited reports on the heterogeneous nucleation-induced CET events in AM-processed Ti alloys, Bermingham et al. [6] employed La_2_O_3_ particles to promote the CET during WAAM and achieved a significant grain size reduction as compared with a Ti-6Al-4V alloy. Barriobero-Vila et al. [43] exploited the peritectic reaction between Ti and La to produce equiaxed β-Ti grains during L-PBF of a Ti-2La alloy. Recently, unmelted metallic particles, such as W, Mo and Nb [44], were also found to act as potent nucleants in the WAAM of Ti-6Al-4V and significantly promote the CET. It has also been reported by Kennedy et al. [45] that during WAAM, adding an appropriate amount of TiN particles into a Ti-6Al-4V alloy could promote the CET and achieve a grain refinement due to an enhanced heterogeneous nucleation. It is noted that it is almost impossible to visualize the microstructural evolution in melt pools during AM through experimental approaches. Thus, mathematical modeling has been conducted recently to investigate the thermal distribution and microstructural evolution during AM. For example, Chen et al. [46] conducted a multi-scale simulation study based on the powder bed model for grain growth during the L-PBF of Inconel 718. The simulation results were consistent with the experimental results in terms of molten pool morphology, grain structure and average grain size [46]. Zinovieva et al. [47,48] proposed a 3D cellular automata finite difference approach to investigate grain structure evolution during the L-PBF of 316L. Yang et al. [49] proposed a phase field model to simulate the grain evolution in the L-PBF of a TiB_2_/316L composite. Their work suggests that TiB_2_ particles act as nucleation sites leading to the formation of equiaxed grains [49].

In this study, ZrN particles will be introduced into a β titanium alloy which will then be processed using L-PBF with the aim of promoting the CET in this alloy. A previous study indicated that ZrN inoculation in a β titanium alloy during casting led to the in situ formation of TiN particles, which provided potent heterogeneous nucleation sites for β-Ti grains, and to strong segregation of Zr along β–Ti grain boundaries [50], both promoting the CET and grain refinement. It is not clear whether the addition of ZrN particles can be equally effective in promoting the CET and grain refinement during the L-PBF of a β titanium alloy and, if the effect is positive, whether the same grain refinement mechanisms would be reproduced during L-PBF. Both an experimental design and mathematical modeling will be carried out to understand the role of ZrN particles in microstructural development in the current L-PBF-processed titanium alloy.

## 2. Materials and Methods

In this work, both experimental and numerical simulation work were carried out to understand the microstructural evolution mechanism of the titanium alloy with the addition of ZrN during L-PBF.

### 2.1. Sample Preparation

**Figure 1:**
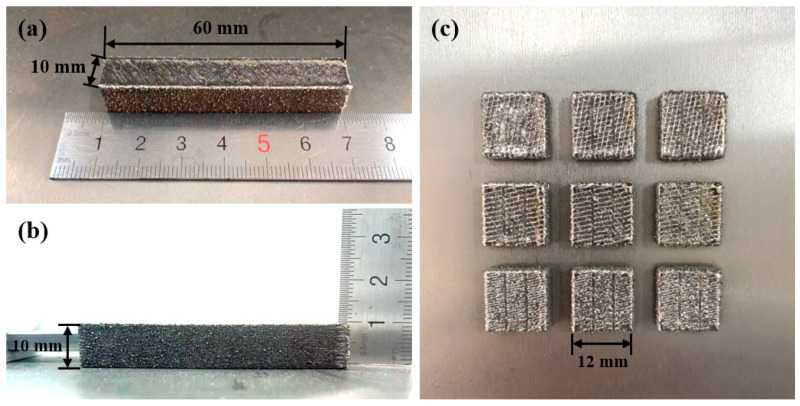
Photos showing the shapes and dimensions of (**a**,**b**) the horizontally built sample and (**c**) single-track laser scanned samples showing single-track melted material on the uppermost surface.

Argon atomized Ti-10V-2Fe-3Al (Ti1023) powder with a size range of 53–105 μm was used in the current study. Its chemical composition was measured using inductively coupled plasma optical emission spectroscopy (ICP-OES), with the carbon content being analyzed using infrared absorption method while the oxygen, nitrogen and hydrogen levels were measured using pulsed-heated inert gas fusion thermal conductivity method. The measured composition is summarized in Table 1. Prior to L-PBF, the as-received alloy powder particles were mixed with 7 wt.% ZrN powder particles which had an average diameter of 1.5 μm. The powder mixing was conducted by using a horizontal cylindrical ball mill which was composed of an engine, a horizontal cylindrical chamber, a shaft along the central axis of the chamber and a number of alternate steel blades which were welded onto the shaft. Powder mixing was realized through the high speed rotation of the blades which brought steel balls and powder particles into air for mixing. Detailed description on the working principle of the current horizontal cylindrical mill can be referred to in [51]. The milling process took 60 min with a shaft rotation speed of 600 rpm. Both pure Ti1023 powder and Ti1023 + 7 wt.% ZrN mixed powder were then processed using a Renishaw AM 400 L-PBF system (Renishaw Ltd., Gloucestershire, UK) which was equipped with an ytterbium fiber laser and has a spot size of 70 μm in diameter. A Ti-6Al-4V substrate which had been thermo-mechanically processed and annealed was used for building samples. The photos of the as-printed samples are shown in Figure 1. Horizontal samples with a dimension of 60 × 10 × 10 mm^3^ (see Figure 1a,b) were fabricated under a modulated pulsed laser mode at a constant laser power of 400 W with a pulsed laser exposure duration of 150 μs. A meander scanning strategy was used for hatch scanning on each layer; after hatch scanning, contour scanning was conducted; see Supplementary Figure S1 [29]. After each layer, the scanning direction was rotated by 67°. A powder layer thickness of 60 μm and a point distance and a hatch distance of 60 μm were used for the current sample preparation. The moving speed of laser exposure from one point to another adjacent point was set to be 5000 mm/s for all of the sample fabrication. The focal length was 450 mm. To better understand the microstructural development during L-PBF processing, particularly the influence of thermal cycling on microstructural development, single track laser scanning at 400 W with exposure durations of 150 μs was conducted. Specifically, bulk samples were built up to 5 mm height first using 400 W and 150 μs, followed by single track laser scanning on the last powder layers; see Figure 1c. By observing the cross sections of these single laser melted tracks, we were able to understand the microstructural development within a single melt pool.

### 2.2. Microstructural Characterization

The as-fabricated samples were longitudinally sectioned using EDM (electrical discharge machining) and then ground using SiC papers from 200 grit up to 4000 grit before being polished using 3 μm diamond suspension and then colloidal silica suspension (or OPS solution). The as-polished samples were then investigated using EBSD (electron backscattered diffraction) and X-ray diffraction (XRD) to understand the grain structure/texture and phase structure development, respectively. The samples were then etched in a solution containing 50 mL distilled water, 25 mL HNO_3_ and 5 mL HF for microstructural characterization using OM (optical microscopy) and SEM (scanning electron microscopy). A Leica DM4000 OM machine (Leica, Wetzlar, Germany), a Zeiss SUPRA55 SEM microscope (Zeiss, Jena, Germany) and a JEOL JSM-7900F SEM microscope (JEOL, Tokyo, Japan) which was fitted with an EBSD detector were used for related microstructural characterization. The step size used for EBSD of the as-fabricated Ti1023 and Ti1023 + 7 wt.% ZrN samples was 2 μm and 1 μm, respectively. It should be noted that the step size was determined according to the grain size in different samples. A Zeiss Gemini300 SEM microscope (Zeiss, Jena, Germany) which was fitted with an EDX (energy dispersive X-ray spectroscopy) detector was used for elemental distribution analysis. TEM study was also performed on some of the as-fabricated samples. Disc specimens with 3 mm in diameter were machined out of the samples and ground into a thickness of 80−110 μm using 800–1200 grit silicon carbide papers and then electro-polished to perforation using a twin-jet electropolisher (RL-1). A polishing solution containing 5% perchloric acid and 95% ethanol was used to electropolish the samples. The TEM experiments were carried out at an accelerating voltage of 200 KV using an FEI TecnaiG20 FEG TEM microscope (Thermo Fisher Scientific, Waltham, MA, USA).

Micro-hardness measurement was also performed on as-fabricated samples by using a Future-Tech FM-800 Vickers (Future-Tech Corp., Tokyo, Japan) diamond indentation instrument. A 300 g force load with a holding time of 15 s was applied for each measurement.

### 2.3. Mathematical Modeling

Since microstructural development is usually dictated by thermal distribution and melt flow behavior in melt pools, mathematical modeling was thus conducted. A computational fluid dynamics (CFD) calculation using Ansys Fluent 19.2 software was carried out. A laser heat source which has a typical Gaussian distribution of heat flux was used in the current modeling. It is defined as follows [52]:

(1) $$Q_{l}\left(x,y,z\right)=\frac{2AP}{\pi R^{2}d}\exp[-2\frac{{\left(x-x_{0}\right)}^{2}+{\left(y-y_{0}\right)}^{2}}{R^{2}}]\exp\left(-\frac{\left|z-z_{0}\right|}{d}\right)$$

where *Q_l_* is the intensity of laser heat source; *A* is the laser absorptivity of powder; *P* is the laser power; *R* is the laser beam spot radius, which was set as 35 μm in the current case; *x*_0_, *y*_0_ and *z*_0_ correspond to *x*-, *y*- and *z*-coordinates of the center of the laser spot projected on the powder bed, respectively; and *d* is the laser penetration depth.

Firstly, for simulation of temperature fields in melt pools, energy input from the heat source and heat loss due to conduction, convection and radiation were taken into account and simulated. The spatial distribution of transient heat transfer during the current pulsed laser melting can be expressed as [53]

(2) $$\rho c\frac{\partial T}{\partial t}=\frac{\partial }{\partial x}\left(k_{t}\frac{\partial T}{\partial x}\right)+\frac{\partial }{\partial y}\left(k_{t}\frac{\partial T}{\partial y}\right)+\frac{\partial }{\partial z}\left(k_{t}\frac{\partial T}{\partial z}\right)+Q_{l}-q_{c}-q_{r}$$

where *ρ* is the density of melt, *c* is the specific heat capacity, *T* is temperature, *t* is time, *k_t_* is the thermal conductivity, *q_c_* is the heat loss due to convection and *q_r_* is the heat loss caused by radiation.

The thermal convection boundary condition was applied to the boundary between the alloy and argon and is expressed as [1,53]

*q_c_*(*x*,*y*,*z*) = *h_c_*(*T* − *T_a_*) (3)

where *h_c_* is the convection heat transfer coefficient and *T_a_* is the ambient temperature, which was measured to be 313 K.

The thermal radiation boundary condition was also exerted on the boundary between the alloy and argon and is expressed as [1,53]

*q_r_*(*x*,*y*,*z*) = *δε*(*T*^4^ − *T_a_*^4^) (4)

where *δ* is Stefan–Boltzmann constant, which is 5.67 × 10^8^ W/(m^2^ K^4^), and *ε* is radiation emissivity which was set as 0.8 in the current study [53].

For simulation of melt flow in a melt pool, gravity, surface tension, Marangoni force and buoyancy force were considered as the main driving forces dictating the melt flow behaviour and thus were all taken into account and simulated. The melt in the pool was regarded as a Newtonian fluid and was assumed to be incompressible and laminar. In order to rationalize the thermal fluid flow, one needs to solve the coupling of the mass conversation equation, momentum conservation equation and energy conservation equation. In the Cartesian coordinate system, the mass conservation equation can be expressed as [1,53,54,55]

(5) $$\nabla \cdot \left(\rho u\right)+\frac{\partial \rho }{\partial t}=0$$

where *u* is the velocity vector.

The momentum conservation equation can be expressed as

(6) $$\frac{\partial \left(\rho u\right)}{\partial t}+\nabla \cdot \left(\rho uu\right)=-\nabla p+\rho g+\nabla \cdot \left(\mu \nabla u\right)+S_{m}$$

where *p* is the pressure, *g* is gravitational acceleration, *μ* is dynamic viscosity and *S_m_* is the momentum source term, which can be expressed as follows:

(7) $$S_{m}=f_{st}+f_{M}+f_{b}$$

where the first term *f_st_* denotes the surface tension force on the gas–liquid interface and is defined as

(8) $$f_{st}=2\sigma \left|\nabla \alpha_{L}\right|\frac{\rho_{\alpha }\kappa }{\left(\rho +\rho_{gas}\right)}$$

(9) κ=−∇⋅n

(10) $$n=\frac{\nabla \alpha_{L}}{\left|\nabla \alpha_{L}\right|}$$

where *σ* is surface tension, *ρ_α_* is the volume-averaged density, *κ* is the curvature of liquid-gas interface, *α_L_* is the liquid phase volume fraction, *ρ_gas_* is density of the argon gas and *n* is the unit normal vector at the liquid–gas interface, which is the unit normal vector of the tangent plane unique to each point on the surface. 

The liquid volume fraction (*α_L_*) of molten metal between solidus and liquidus points is assumed to vary with temperature linearly. The relationship between liquid volume fraction and metal temperature can be defined as [53]

$$\alpha_{L}=0,\;\;\;\;\;\;\;\;\;\;\;\;\;\;\;\;\;\;\;\;\;\;T<Ts$$

(11) $$\alpha_{L}=\frac{T-T_{S}}{T_{L}-T_{S}},\;Ts\leq T\leq T_{L}$$

$$\;\alpha_{L}=1,\;\;\;\;\;\;\;\;\;\;\;\;\;\;\;\;\;\;\;\;\;T>T_{L}$$

where *T_S_* is the solidus temperature and *T_L_* is the liquidus temperature.

In Equation (7), the second term *f_M_* is the Marangoni shear force caused by surface tension gradient, which can be expressed as

(12) $$f_{M}=2\frac{d\sigma }{dT}\left[\nabla T-\left(n\cdot \nabla T\right)n\right]\left|\nabla \alpha L\right|\frac{\rho_{aL}}{\left(\rho_{liquid}+\rho_{gas}\right)}$$

where *dσ*/*dT* is Marangoni coefficient, which describes the temperature coefficient of surface tension. In the molten pool, the Marangoni flow caused by thermal gradient can affect the mass and heat transfer and thus the temperature field, velocity field and molten pool morphology [1,53,54].

The third term of *S_m_* in Equation (7) is the buoyancy. According to Boussinesq approximation, the buoyancy force produced by density difference due to thermal expansion can be described as

(13) $$f_{b}=\rho g\beta \left(T-T_{ref}\right)$$

where *β* is thermal expansion coefficient, *T* is the melt metal temperature and *T_ref_* is the reference temperature, equal to 313 K in this case.

The energy conservation equation is shown below:

(14) $$\frac{\partial \left(\rho H\right)}{\partial t}+\nabla \cdot \left(\rho uH\right)=\nabla \cdot \left(k_{t}\nabla T\right)+S_{h}$$

where *H* is the enthalpy of the material, *k_t_* is thermal conductivity and *S_h_* is the energy source term which includes two parts, heat exchange with the ambient and release of the latent heat during melting. Thus, *S_h_* is defined as

(15) $$S_{h}=Q_{l}-q_{m}-q_{c}-q_{r}$$

where *q_m_* is the heat loss due to melting of the metallic material.

For modeling, a cuboid geometric model with a dimension of 600 × 500 × 500 mm^3^ was established in Ansys Fluent, and the melt pool region was meshed with smaller elements (containing 86,400 hexahedral cells) to enhance the precision of melt flow simulation. Supplementary Figure S2 shows the boundary conditions set in the simulation model. A laser power of 400 W and an exposure duration of 150 μs, which give rise to an energy density of 277.8 J/mm^3^, were used for the current simulation of melt pools. The temperature-dependent density, thermal conductivity and specific heat capacity of the titanium alloy, which were obtained via calculation using the Jmatpro 7.0 software, are listed in Table 2. The constant physical properties used in the simulation are shown in Table 3.

## 3. Results

**Figure 2:**
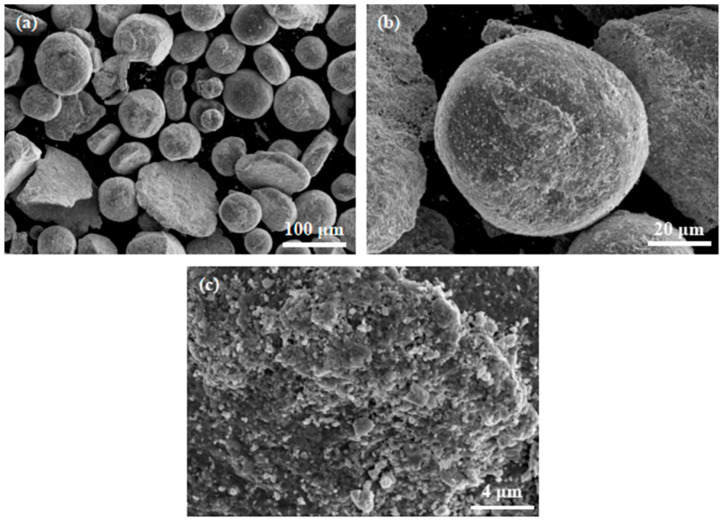
SEM micrographs of Ti1023 + 7 wt.% ZrN powder particles after ball milling for 60 min. (**a**) The particle distribution in the mixed powder; (**b**,**c**) the distribution of ZrN particles on the surfaces of large Ti1023 particles.

Figure 2 shows the morphology and surface structure of Ti1023 + 7 wt.% ZrN mixed powder particles. It is clear that while many Ti1023 alloy powder particles still remained near-spherical after ball milling, many other particles have obviously been flattened and show a disc-like morphology. The added ZrN particles were found to homogenously distribute and decorate on the alloy powder particle surfaces and seem to have developed a good bonding with the alloy particles. This indicates that the current ball milling method is effective in homogenously distributing ZrN particles among the alloy powder particles.

**Figure 3:**
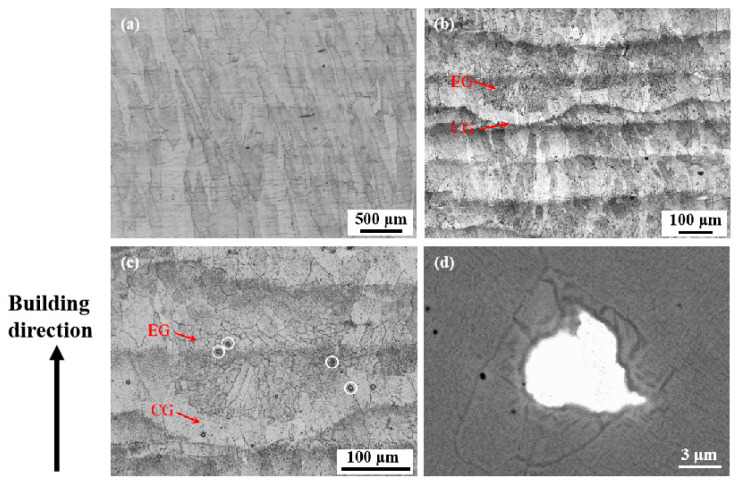
OM micrographs of the grain structures in (**a**) Ti1023 and (**b**,**c**) Ti1023 + 7 wt.% ZrN samples that were made with an exposure duration of 150 μs; (**d**) backscattered electron SEM micrograph of one of the particles indicated in (**c**) by loops. EG and CG in (**b**) and (**c**) stand for equiaxed grains and columnar grains, respectively.

Figure 3 shows the OM micrographs of grain structures in the as-fabricated Ti1023 and Ti1023 + 7 wt.% ZrN samples. It is obvious that the as-fabricated Ti1023 sample was dominated by large columnar grains, many of which extended through many layers along the building direction (see Figure 3a). With the addition of 7 wt.% ZrN, the grain structure changed significantly. The as-fabricated Ti1023 + 7 wt.% ZrN sample consisted of alternate layers of equiaxed grains and columnar grains, indicating that a competitive development of equiaxed grains and columnar grains may have happened during solidification after L-PBF. Moreover, it is noted that there are a number of horizontal black bands with a spacing of around 100 μm throughout the as-fabricated samples. These bands tended to penetrate through equiaxed grain zones and seem to be decorated with a high density of fine particles (Figure 3b,c). The chemical composition (at. %) of the particle shown in Figure 3d obtained using SEM-EDX analysis is listed in Table 4, which indicated that this particle is ZrN.

**Figure 4:**
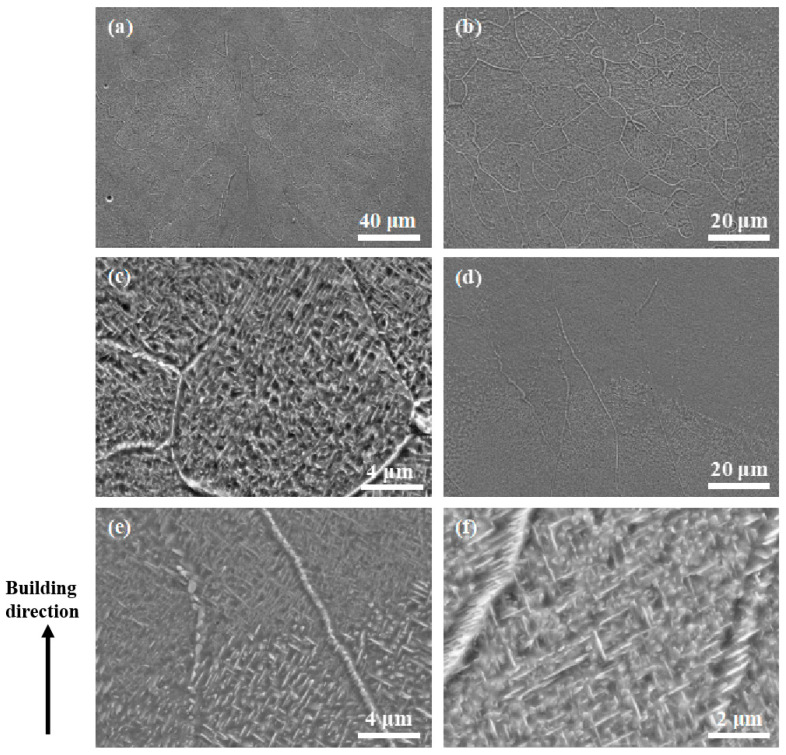
SEM micrographs showing (**a**) a band structure and its adjacent regions in the Ti1023 + 7 wt.% ZrN sample made with a pulsed laser exposure duration of 150 μs; (**b**) the grain structure within the band; (**c**) microstructure in an equiaxed grain within the band; (**d**) a transition region across the boundary of the band structure; (**e**) microstructure across the boundary of the band structure; (**e**,**f**) microstructure of part of a columnar grain which is outside the band structure.

**Figure 5:**
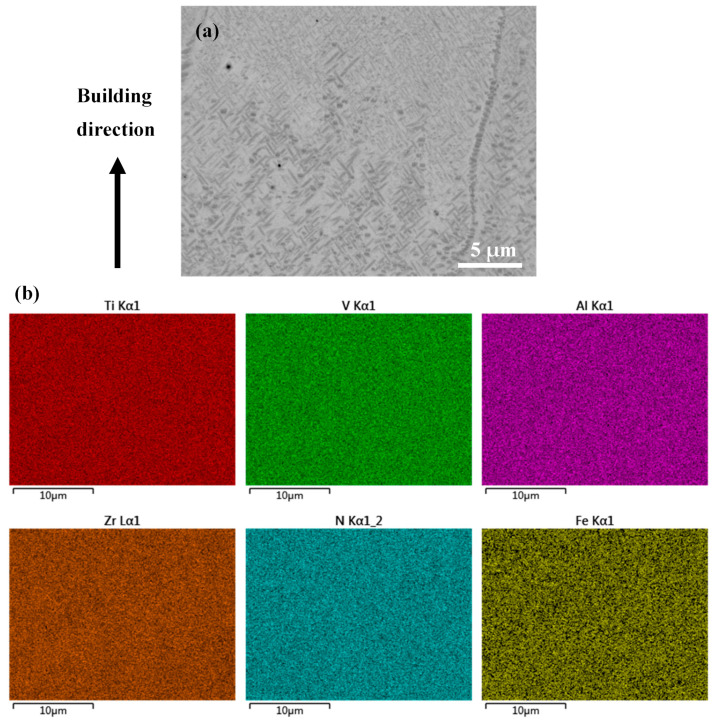
(**a**) Backscattered electron SEM micrograph and (**b**) EDX mapping on a transition region across the boundary of a band structure in the as-fabricated (Ti1023 + 7 wt.% ZrN) sample in polished state.

To fully understand the band structure, SEM was employed to study the microstructure. Under secondary electron SEM, the band structures turned bright as compared with their surrounding regions (see Figure 4a,b). Consistent with the above OM observation, the band structures were dominated by equiaxed grains. Grains within these bands, whether equiaxed or columnar, were found to contain lamellar precipitates that were thicker than those outside the bands (Figure 4c–f). Quantitative measurements based on the SEM micrographs revealed that the average thickness of the laths within the bands was approximately 150 nm, which is in contrast to the average thickness of 75 nm for those outside the bands. This phenomenon suggests that the band regions may either have experienced a thermal history that is different from their adjacent regions or have chemical segregation. To clarify this issue, EDX mapping was performed on a transition region across the boundary of a band structure (see Figure 5a). The results, as shown in Figure 5b, demonstrate that the transition region showed a good chemical homogeneity, suggesting that the formation of the band structures which contain coarser lamellar precipitates should not be due to chemical segregation but instead could be due to a unique thermal history.

**Figure 6:**
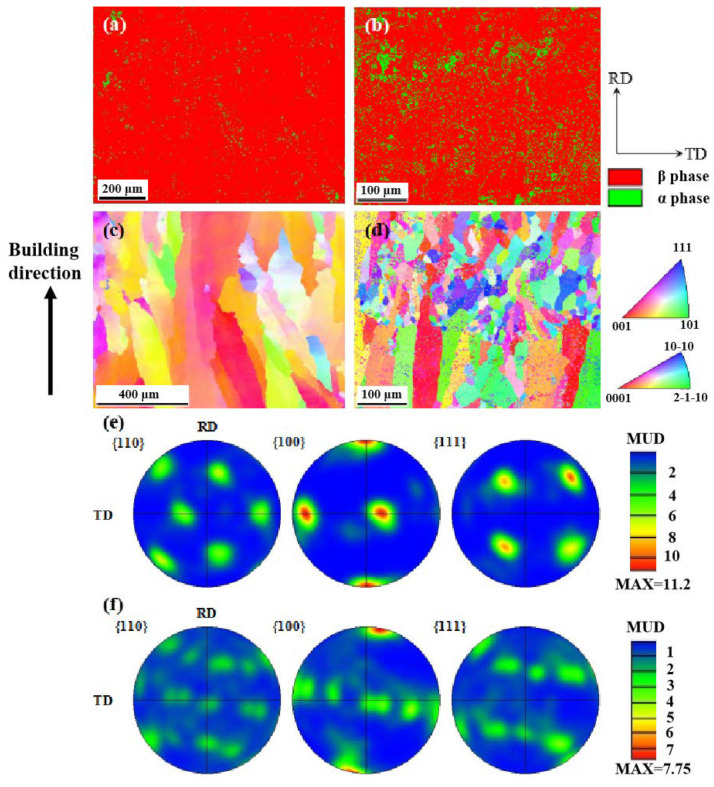
(**a**,**b**) EBSD phase maps and (**c**,**d**) IPF (inverse pole figure) color maps of L-PBF-processed Ti1023 (left column) and (**b**) Ti1023 + 7 wt.% ZrN (right column); EBSD pole figures of β phase in L-PBF-processed (**e**) Ti1023 and (**f**) Ti1023 + 7 wt.% ZrN. The area fraction of α in (**a**) and (**b**) is 0.4% and 12.7%, respectively. RD is parallel to the building direction.

**Figure 7:**
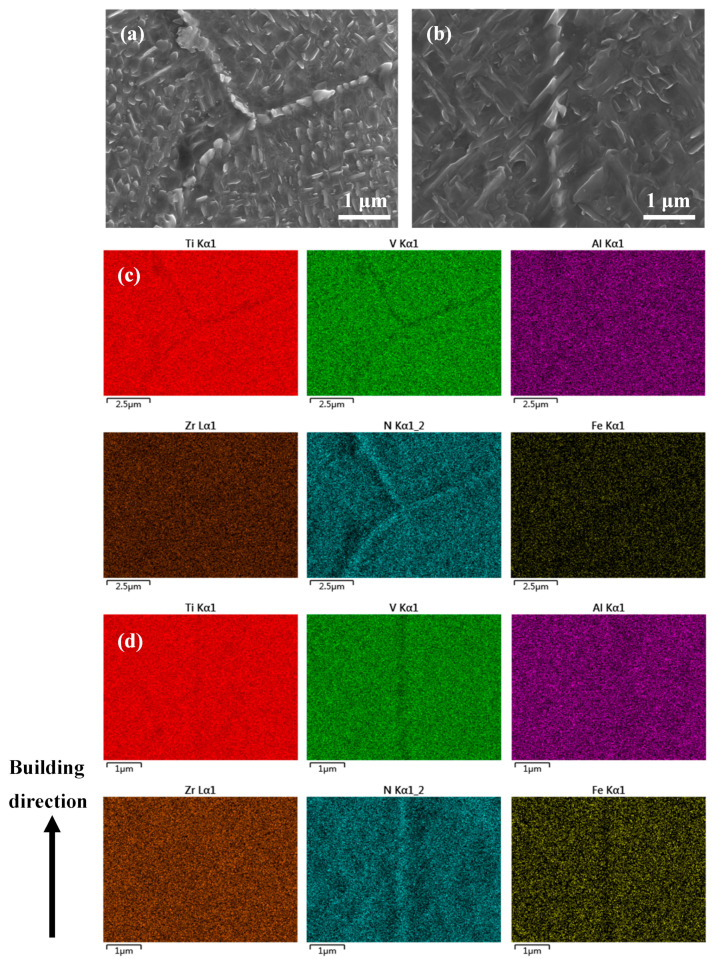
High magnification (**a**,**b**) SEM micrographs and (**c**,**d**) EDX mapping on grain boundary regions of both (**a**,**c**) equiaxed grains and (**b**,**d**) columnar grains in the L-PBF-processed Ti1023 + 7 wt.% ZrN sample. The arrow in (**a**) points to the site which was analyzed using point EDX shown in Table 5.

EBSD was further employed to study the microstructure of as-fabricated samples. According to the phase maps shown in Figure 6, the as-fabricated Ti1023 + 7 wt.% ZrN sample was composed of α and β phases. Thus, the lamellar precipitates within grains (Figure 4) could be determined as α laths. It is worth mentioning that the sample without ZrN addition also contained a small fraction of α, which was approximately 0.4%. With the addition of 7 wt.% ZrN, the area fraction of α increased significantly to 12.7%. High-magnification SEM micrographs and EBSD mapping showed that the α phase was located not only within grains but also along grain boundaries (GBs) (see Figure 4c–f and Figure 6a,b). The α phase along the GBs was either continuous or discontinuous. EBSD pole figures (Figure 6c,d) show that the α phase obeyed the Burgers orientation relationship with the β matrix. To understand the formation mechanism of α along GBs, high magnification SEM and EDX mapping were employed to investigate the chemical distribution in the grain boundary regions of both equiaxed grains and columnar grains (see Figure 7). Meanwhile, the chemical composition at the grain boundary of the as-fabricated Ti1023 + 7 wt.% ZrN alloy is listed in Table 5 according to the EDX results. The results indicate that the GBs were enriched in N. Given that N is an alpha stabilizer, the enrichment in N promoted the development of α along the GBs.

**Figure 8:**
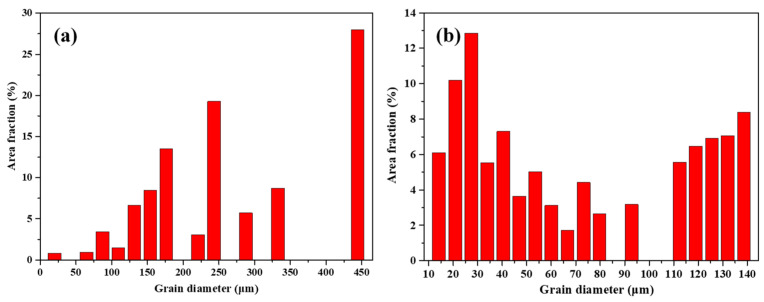
Grain size distribution obtained from the EBSD analysis on L-PBF-processed (**a**) Ti1023 and (**b**) Ti1023 + 7 wt.% ZrN.

Based on the EBSD results shown in Figure 6c–f, the L-PBF-processed Ti1023 sample demonstrates monotonic large columnar grains, which led to a high texture along the <001> direction. The L-PBF-processed Ti1023 + 7 wt.% ZrN sample shows combined equiaxed and columnar grains (Figure 6d), which is in good agreement with the above OM and SEM observations. With the presence of equiaxed grains, the texture level was remarkably decreased, as evidenced by the decrease in the maximum MUD value (Multiple of the Uniform Distribution) from 11.2 in the L-PBF-processed Ti1023 to 7.75 in the L-PBF-processed Ti1023 + 7wt.% ZrN sample (Figure 6e,f). Similarly, the grain distribution changed significantly. For the L-PBF-processed Ti1023 sample, a vast majority of grains were larger than 100 μm in diameter, whereas in the L-PBF-processed Ti1023 + 7 wt.% ZrN sample, almost all the grains were smaller than or equal to 100 μm in diameter, with a majority of the grains being smaller than 50 μm (see Figure 8). It is thus clear that the addition of 7 wt.% ZrN caused significant grain refinement to the current titanium alloy.

**Figure 9:**
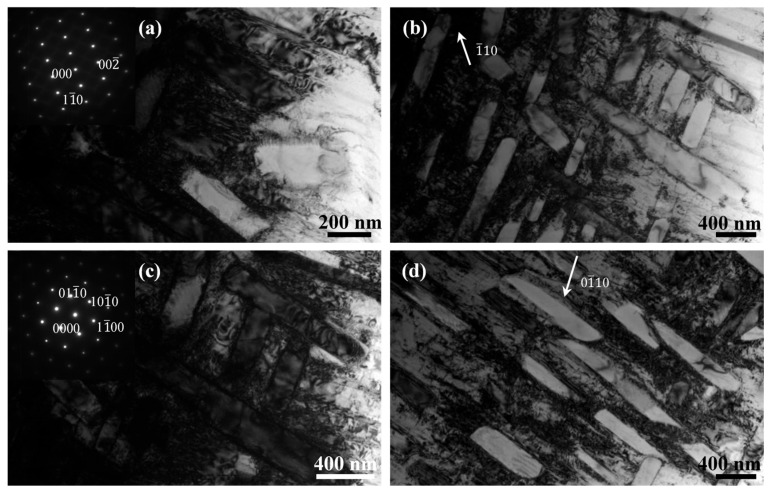
Bright-field(BF) TEM micrographs of L-PBF-processed Ti1023 + 7 wt.% ZrN sample obtained along (**a**) pole [110]β and (**c**) pole [0001]α, respectively; (**b**,**d**) are BF TEM micrographs of the as-fabricated Ti1023 + 7 wt.% ZrN sample obtained under two-beam conditions from (**a**) pole [110]β and (**c**) pole [0001]α, respectively.

Figure 9 shows the TEM micrographs and diffraction patterns of the as-fabricated Ti1023 + 7 wt.% ZrN sample. The selected area diffraction patterns from the matrix and lamellar precipitates further confirmed that the sample is composed of β and α phases (see the insets in Figure 9a,c). Moreover, the TEM micrographs obtained under two-beam conditions based on either the β matrix or α phase demonstrated that there was a high density of dislocations present within the β matrix (Figure 9b,d). In contrast, the lamellar α phase was almost free of dislocations, suggesting that the α phase may have formed through the β→α phase transformation during cooling.

**Figure 10:**
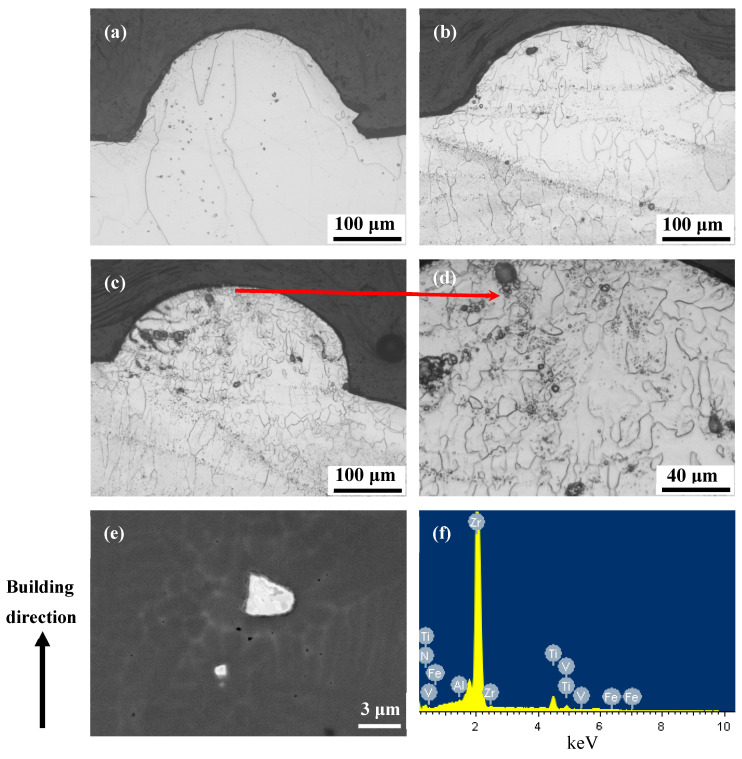
OM micrographs showing the grain structure within a single cross-section of a laser melted track of (**a**) Ti1023 and (**b**–**f**) Ti1023 + 7 wt.% ZrN made with laser exposure duration of 150 μs, (**d**) is an enlarged view of the area (marked by red arrow) of (**c**); (**e**) backscattered electron SEM micrograph showing two particles present in the solidified melt pool indicated in (**d**); (**f**) Point EDX analysis result of the larger particle shown in (**e**).

To understand the mechanism of microstructural development during the L-PBF of the current materials, single track laser melting of powder particles was conducted on the top surfaces (with a powder layer upon them) of some bulk L-PBF-processed samples using different laser exposure durations. Figure 10 shows the microstructures of some cross- sections of the solidified tracks. These sections actually correspond to longitudinal sections of solidified melt pools and thus are very useful for understanding the microstructural evolution during L-PBF. It is clear that for the L-PBF-processed Ti1023, the single solidified melt pool was fully dominated by several large columnar grains which grew epitaxially from a previous layer, whereas for the L-PBF-processed Ti1023 + 7 wt.% ZrN, the solidified melt pools tended to show columnar grains in the lower regions but fine equiaxed grains in the upper regions. Obviously, columnar grains seem quite liable to be developed at the interlayer interfaces due to epitaxial grain growth. Moreover, it is noted that a number of particles accumulated in the upper regions of the solidified melt pools of the L-PBF-processed Ti1023 + 7 wt.% ZrN sample (see Figure 10b–d). These particles, according to the point EDX analysis (Figure 10e,f), were enriched in Zr and N, and the chemical compositions of some particles in the as-fabricated Ti1023 + 7 wt.% ZrN alloy are listed in Table 6 according to the EDX results, suggesting that they could be unmelted ZrN particles.

**Figure 11:**
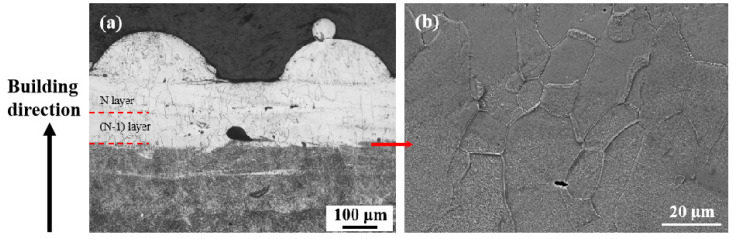
(**a**) OM micrograph showing the microstructure in the last several layers of L-PBF-processed Ti1023 + 7 wt.% ZrN made with an exposure duration of 150 μs; (**b**) SEM micrograph showing the microstructure at an interlayer boundary area indicated by the arrow in (**a**). N layer corresponds to the last layer of the build.

In addition, it is worth mentioning that the last two layers of the as-fabricated Ti1023 + 7 wt.% ZrN sample showed a sharp contrast to the previous layers under OM observation (Figure 11a). SEM imaging revealed that the last two layers were dominated by a single β phase, whereas the previous layers consisted of both β grains and massive fine precipitates (Figure 11b). That the last two layers did not undergo repeated thermal cycles during L-PBF could account for the single β phase in this region.

With the addition of 7 wt.% ZrN, the microhardness of the alloy was found to have been significantly improved. In general, the L-PBF-processed Ti1023 samples made at 150 μs showed an average Vickers hardness of 321 ± 8 HV, whereas the L-PBF-processed Ti1023 + 7 wt.% ZrN samples made at 150 μs exhibited an average hardness of 493 ± 22 HV in equiaxed grains and 493 ± 21 HV in columnar grains. This means that an average 172 HV improvement in microhardness was achieved after the addition of ZrN, which could be attributed to the formation of a high density of ultrafine α precipitates, the additional solid solution strengthening from solute Zr and refinement in grain structure [50].

## 4. Discussion

Based on the above experimental observations, both the grain structure and phase constituent of the titanium alloy changed with the addition of ZrN. In this section, the microstructural evolution mechanism during L-PBF with the addition of ZrN is discussed.

### 4.1. On the Origin for Competitive Columnar–Equiaxed Grain Development with the Addition of ZrN

The current experimental results have clearly demonstrated that the addition of 7 wt.% ZrN to Ti1023 remarkably promotes the CET process, particularly in the upper regions of the solidified melt pools, which were dominated by equiaxed grains. The lower regions of the melt pools or interlayer boundary regions, however, were still dictated by columnar grains. It is obvious that the addition of ZrN has a significant influence on the microstructural development in the current material. The formation of equiaxed grains in the upper regions of melt pools is believed to be a result of multiple effects from solutes such as N and unmelted ZrN particles.

**Figure 12:**
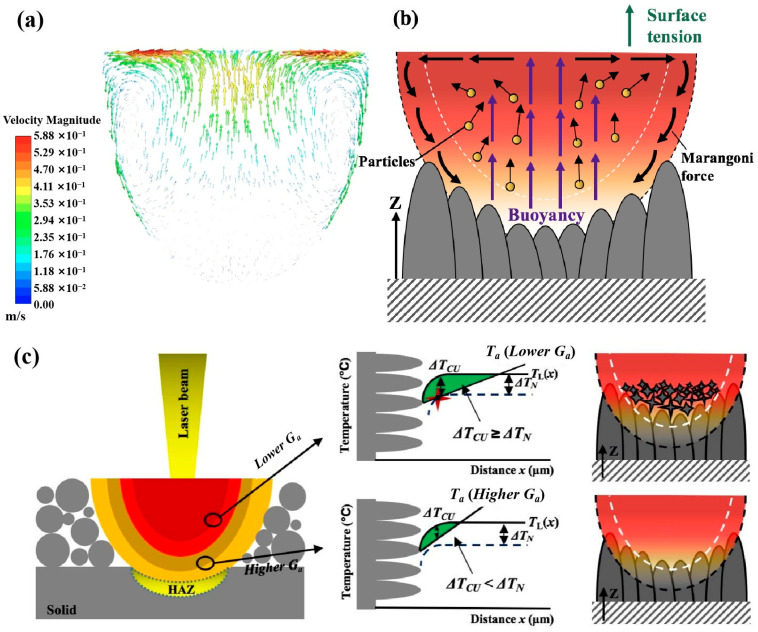
(**a**) Mathematical modeling results showing the melt flow behavior within a melt pool; (**b**) schematic illustration showing different forces involved in different melt flow behaviors within a melt pool and their influence on the movement of unmelted particles; (**c**) schematic illustration of thermal gradient distribution and different solidification processes in different regions of a melt pool. HAZ stands for heat-affected zone; *G_a_* stands for thermal gradient; *T_a_* stands for actual temperature; *T_L_* stands for liquidus temperature; ∆*T*_CU_ stands for constitutional undercooling; ∆*T*_N_ stands for the critical amount of undercooling required for equiaxed grain nucleation.

It is well known that a sufficient amount of CU in the liquid ahead of the solidification front is essential for nucleation. The condition required for the existence of the CU zone is that the actual temperature (*T_a_*) gradient, *G_a_*, at the interface in the liquid should be lower than the gradient of liquidus temperature (*G_L_*) change in the melt (illustrated in Figure 12c), which is obtained by multiplying the concentration gradient, *G_c_*, by the liquidus slope, *m*. Therefore, the interface is constitutionally undercooled when [56]

*G_a_* < *mG_c_* (16)

**Figure 13:**
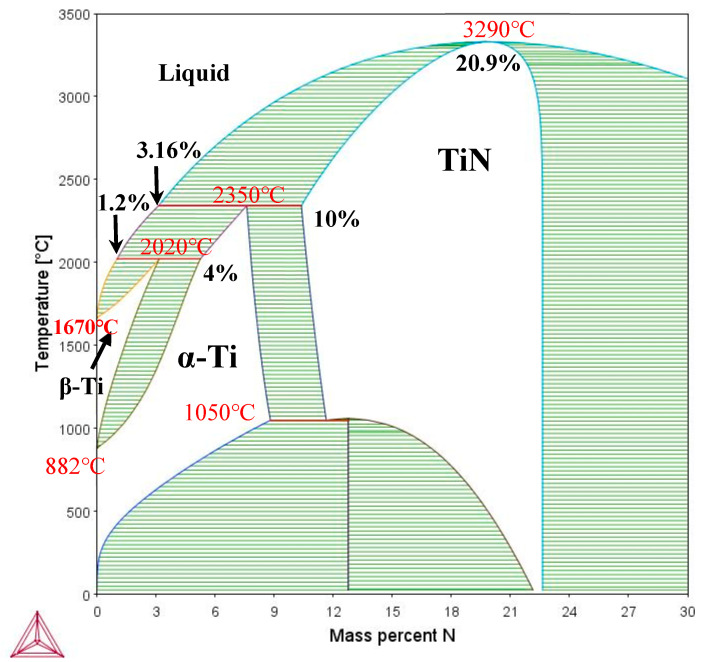
Calculated Ti-N phase map.

Since $G_{c}=-\left(\frac{V}{D}\right)\Delta C_{0}$, where $\Delta C_{0}=\frac{C_{0}\left(1-k\right)}{k}$ and *V* and *D* are the grain growth rate of the solid/liquid interface and the diffusion coefficient of solute atoms, respectively. *k* is the partition coefficient. The criterion for the existence of CU can be written in another form:

(17) $$G_{a}<-\frac{mV\Delta C_{0}}{D}\;\mathrm{or}\;\frac{G_{a}}{V}<\frac{mC_{0}}{D}\cdot \frac{1-k}{k}\;$$

where *Q* = *mC*_0_(1 − *k*) is often defined as the growth restriction factor. A larger *Q* means that the rate of forming CU is faster [16]. It is noted that interstitial elements, such as C and O, all show a high *Q* [16]. The *m* and *k* values of N in the present alloy were also calculated based on the measurement of N in the as-fabricated sample, which was around 0.86 wt.%, and the Ti-N phase map which is shown in Figure 13. The value of the liquidus slope, *m* = 179, and partition coefficient, *k* = 10, can be obtained from the Ti-N binary phase diagram. Based on the calculation, the *Q* value of N in the alloy was also derived. It is obvious that both the *m* and *Q* of N are extraordinarily large. Given that N has the largest *m* among all the solutes for the current alloy, its dissolution in the melt would obviously elevate the gradient of liquidus temperature (*mG_c_*), which means that ∆*T*_CU_ would be expanded even if the actual melt gradient *G_a_* remained the same. The large *Q* of N, according to Formula (17), also indicates that the CU zone could be developed much faster ahead of the solidification front, which would be beneficial for nucleation and grain refinement. The observation of the segregation of N along grain boundaries (Figure 7) further confirms that N can indeed restrict the grain growth and is benign to grain refinement.

Moreover, it was noted that while the majority of ZrN particles were fully melted and dissolved in the metallic matrix, a small population of them still remained unmelted or partially melted and tended to accumulate in the upper regions of the solidified melt pools. This phenomenon is believed to be the result of melt flow behavior within each melt pool. Our modeling results in Figure 12a,b show that under surface tension and Marangoni force, the melt on the top surface tends to move outwards from the centre and in the lateral subsurface regions the melt would move downwards towards the bottom regions. Meanwhile, in the middle regions of the melt pool, melt tends to move upwards under the drive of the buoyance force. This type of melt flow behavior would obviously help bring the unmelted particles near the solid–liquid interface up to the upper regions and promote the accumulation of unmelted particles in the upper regions. According to the edge-to-edge theory [57,58,59] and research by Kennedy et al. [45], ZrN can be a potent nucleant for Ti alloy. Gäumann et al. [60] developed a relationship between the thermal conditions and the volume fraction of equiaxed grains, *φ*, which can be given by

(18) $$\emptyset =1-\exp\left[-\frac{4\pi N_{0}}{3}{\left(\frac{\Delta T_{\mathrm{CU}}}{G\left(1+n\right)}\right)}^{3}\right]$$

where *G* is the temperature gradient in the liquid, *n* is materials dependent constant and *N*_0_ is the number of nucleation sites. According to Formula (18), the addition of the nucleant can increase the number of nucleation sites, *N*_0_, so that the volume fraction of equiaxed grains increases. A considerable amount of unmelted ZrN particles, which exist in the upper regions of melt pools, would thus undoubtedly offer sufficient heterogeneous nucleation sites to decrease the energy barrier for grain nucleation. This is also beneficial for CET and grain refinement.

In general, the formation of equiaxed grains and grain refinement in the melt pools of the Ti1023 + 7 wt.% ZrN sample could be attributed to multiple factors including an increased gradient of liquidus temperature caused by the N solute, a grain growth restriction caused by N segregation along the grain boundaries and the accumulation of unmelted ZrN particles in the upper regions of the melt pools potentially acting as heterogeneous nucleating substrates. As such, the role of ZrN in the present study is totally different from that observed in a previous study on a cast β titanium alloy where, when ZrN was dissolved in the melt [50], TiN formed and acted as a potent heterogeneous nucleant instead. In the current study, TiN was not observed, which could be because the N level in the liquid ahead of the solidification front was below the critical N level (3.16 wt.%) required for the nucleation of TiN from the liquid (see Figure 13). This is possible given that the bulk as-fabricated Ti1023 + 7 wt.% ZrN sample only contained 0.86 wt.% N.

It is also noted that at the bottom regions or interlayer interfaces of the melt pools, columnar grains still dominated. This could be mainly due to the fact that the lower regions of the melt pools tend to experience relatively higher actual temperature gradients throughout the solidification process. The higher *T_a_* would inevitably reduce the ∆*T*_CU_ and when ∆*T*_CU_ is smaller than ∆*T*_N_, no nucleation of new grains would occur, whereas the lower *T_a_* in the upper regions would increase the ∆*T*_CU_ and promote the nucleation of new grains, as illustrated in Figure 12c. Furthermore, according to Formula (18), a large actual temperature gradient *G_a_* is unfavorable for the formation of equiaxed grains. Moreover, the bottom regions correspond to the early stage of solidification where the solute concentration ahead of the solidification front is still at a low level and, as a result, they may not be able to affect the liquidus temperature gradient or be effective in grain growth restriction. Furthermore, at the beginning of solidification, unmelted ZrN particles in the melt pools are more dispersive and less dense, which cannot promote significant heterogeneous nucleation. Nonetheless, the much steeper melt temperature gradient and lack of unmelted ZrN particles at the lower regions should be the main factor responsible for the development of large columnar grains. In summary, the CET in an L-PBF-processed titanium alloy could be achieved with refractory ceramic powder addition (e.g., ZrN), but a higher added amount is required as compared with WAAM and L-DED.

### 4.2. On the Formation Mechanism of α Precipitates

The current experimental results demonstrated that the as-fabricated Ti1023 + 7 wt.% ZrN sample is dominated by α precipitates. This is in sharp contrast to the as-fabricated Ti1023, where no obvious α precipitates can be observed using OM or SEM. Since the two samples were fabricated under the same processing conditions, the difference in microstructure suggests that it was the addition of ZrN instead of the processing conditions or thermal history that significantly promoted α precipitation. However, on the other hand, it was observed that laser reheating also has a great influence on the microstructural development. The building of a new layer of powder seems to have successfully suppressed α precipitation not only in the newly built layer but also in the previous layer (see Figure 11). This suggests that the laser reheating temperature imposed on the previous layer should have gone beyond the β transus in the Ti1023 + 7 wt.% ZrN, and in the subsequent rapid cooling the β→α transformation would be suppressed. Apart from the last two layers, which were dominated by the β phase, the layer built prior to the last two layers was found to be full of α precipitates, as were the other previous layers. Notably, the 7 wt.% ZrN addition increased the N content of the Ti alloy, which would lead to the formation of precipitates and may decrease the ductility. Optimizing the amount of ZrN and the mechanical behavior will be the focus of future work.

## 5. Conclusions

- The addition of ZrN particles into a beta titanium alloy during L-PBF transforms the microstructure from dominant large columnar grains into mixed equiaxed grains and refined columnar grains. Equiaxed grains tend to form in the upper regions of melt pools while columnar grains form in the lower regions, which, according to the experimental and mathematical modeling results, is mainly due to the accumulation of unmelted ZrN particles and relatively lower thermal gradients in the upper regions.
- Other factors, such as an increased gradient of liquidus temperature caused by N solute and grain growth restriction effect caused by the segregation of N along grain boundaries, may have also contributed to the formation of equiaxed grains in the ZrN-bearing alloy.
- The addition of ZrN results in significant α precipitation which shows strong variant selection tendency. The massive α precipitation was found to be driven by laser reheating and the N solute in the matrix.

## Figures and Tables

**Table 1:** The chemical composition measurement results of as-received gas atomized Ti-10V-2Fe-3Al powder.

Element	V	Fe	Al	O	C	N	H	Ti
wt.%	10.64	1.85	3.30	0.086	0.0053	0.028	0.0036	Bal.

**Table 2:** Temperature-dependent thermo-physical property parameters of Ti1023 used in the current modeling.

Temperature K	Density kg/m^3^	Thermal Conductivity W/(m·K)	Specific Heat Capacity J/(kg·K)
298	4580	7.5	540
468	4530	8.7	580
608	4490	9.9	600
963	4440	13.9	620
1248	4380	16.9	640
1493	4320	19.2	680
1698	4290	24.3	805
1868	4250	22.2	890
1948	4200	26.3	960
1973	4190	34.6	960
2028	4170	35.8	960

**Table 3:** Constant physical property values used in the current finite element analysis.

Physical Property	Value	Reference
Solidus temperature (K)	1878	[55]
Liquidus temperature (K)	1928	[55]
Latent heat of melting (J/kg)	2.86 × 10^5^	[55]
Viscosity (Pa·s)	0.005	[55]
Thermal expansion coefficient (K^−1^)	8 × 10^−6^	[53]
Surface tension (N/m)	1.4	[53]
Temperature coefficient of surface tension (N/m/K)	−2.6 × 10^−4^	[55]
Atmospheric pressure (Pa)	101,300	
Ideal gas constant (J/K/mol)	8.314	
Gravitational acceleration (m/s^2^)	9.8	

**Table 4:** Chemical composition (at. %) of the particle shown in Figure 3d obtained using SEM-EDX analysis.

Element	Zr	N	Ti
at. %	69.07	29.39	1.54

**Table 5:** Chemical composition on the grain boundary of the as-fabricated Ti1023 + 7 wt.% ZrN alloy.

Element	V	Fe	Al	N	Zr	Ti
wt.%	8.14	1.47	2.57	0.79	9.56	Bal.

**Table 6:** Chemical composition (at. %) of the particle shown in Figure 10e obtained using SEM-EDX analysis.

Element	Zr	N	V	Fe	Al	Ti
at. %	58.38	33.69	1.02	0.33	0.38	6.19

## Data Availability

Data are contained within the article and Supplementary Materials.

## Supplementary Materials

The following supporting information can be downloaded at: https://www.mdpi.com/article/10.3390/mi15010104/s1, Figure S1: Schematics showing the hatch scanning strategy and contour scanning (yellow lines) used in the present study; Figure S2: Schematic showing the boundary conditions set in the simulation model of a melt pool.
