# Supplementary material for: On the Role of ZrN Particles in the Microstructural Development in a Beta Titanium Alloy Processed by Laser Powder Bed Fusion
Source: Micromachines (Basel). 2024 Jan 5;15(1):104. doi: 10.3390/mi15010104 (PMC10819300; doi:10.3390/mi15010104)
Supplement: Supplementary file 1 [file micromachines-15-00104-s001.zip › micromachines-2753086-supplementary.pdf]

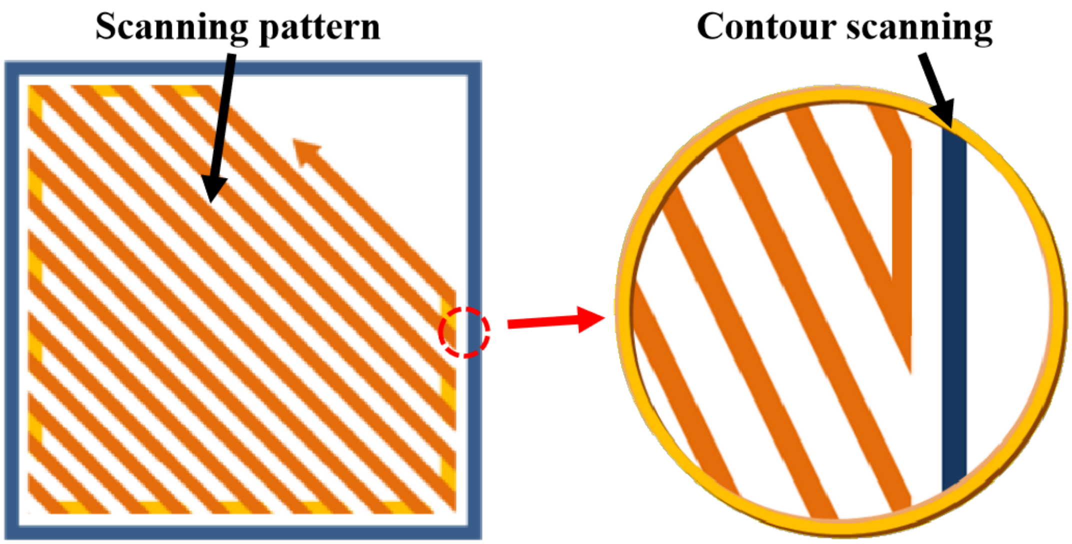

Supplementary Figure S1. Schematics showing the hatch scanning strategy and contour scanning (yellow lines) used in the present study.

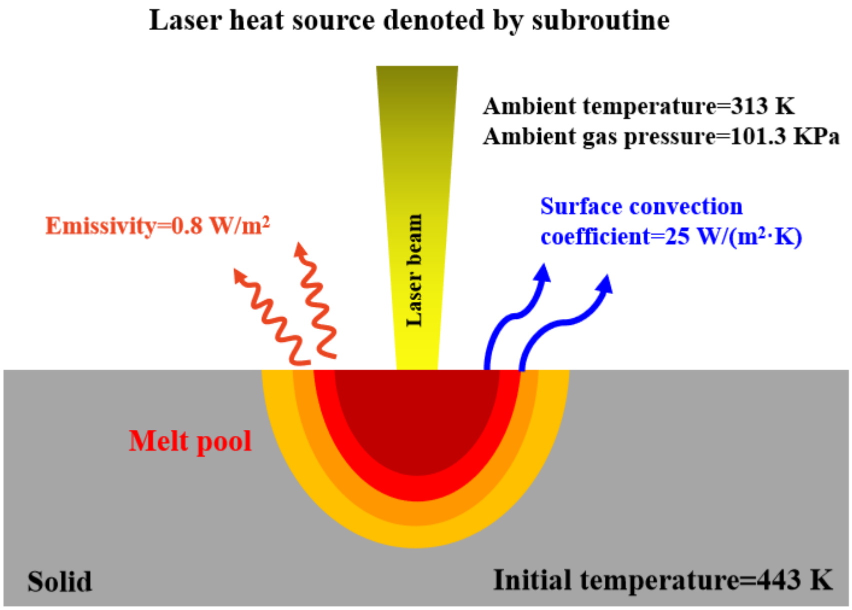

Supplementary Figure S2. Schematic showing the boundary conditions set in the simulation model of a melt pool.
